# Triggering Endogenous Cardiac Repair and Regeneration via Extracellular Vesicle-Mediated Communication

**DOI:** 10.3389/fphys.2018.01497

**Published:** 2018-10-23

**Authors:** Sveva Bollini, Anke M. Smits, Carolina Balbi, Edoardo Lazzarini, Pietro Ameri

**Affiliations:** ^1^Regenerative Medicine Laboratory, Department of Experimental Medicine, University of Genova, Genoa, Italy; ^2^Laboratory of Cardiovascular Cell Biology, Department of Cell and Chemical Biology, Leiden University Medical Center, Leiden, Netherlands; ^3^Laboratory of Molecular and Cellular Cardiology, CardioCentro Ticino, Lugano, Switzerland; ^4^Laboratory of Cardiovascular Biology, Department of Internal Medicine, University of Genova, Genoa, Italy; ^5^Cardiovascular Disease Unit, IRCCS Ospedale Policlinico San Martino, Genoa, Italy

**Keywords:** paracrine effect, stem cell-extracellular vesicles, cardiac progenitor cell, cardiomyocyte, fibroblast, exosome

## Abstract

A variety of paracrine signals create networks within the myocardium and mediate intercellular communications. Indeed, paracrine stimulation of the endogenous regenerative program of the heart, mainly based on resident cardiac progenitor cell (CPC) activation together with cardiomyocyte proliferation, has become increasingly relevant for future cardiac medicine. In the last years, it has been shown that extracellular vesicles (EV), including exosomes (Ex), are powerful conveyors of relevant biological effects. EV have been proposed not only as promising therapeutic tool for triggering cardiac regeneration and improving repair, but also as means of better understanding the physiological and pathological relationships between specific cardiac components, including cardiomyocytes and fibroblasts. Actually, EV from different kinds of exogenous stem cells have been shown to mediate beneficial effects on the injured myocardium. Moreover, endogenous cells, like CPC can instruct cardiovascular cell types, including cardiomyocytes, while cardiac stromal cells, especially fibroblasts, secrete EV that modulate relevant aspects of cardiomyocyte biology, such as hypertrophy and electrophysiological properties. Finally, cardiomyocytes too may release EV influencing the function of other cardiac cell types. Therefore, EV-based crosstalk is thought to be important in both physiology and pathology, being involved in the responses of the heart to noxious stimuli. In this review we will discuss the role of EV in both regulating cardiac homeostasis and driving heart regeneration. In particular, we will address their role in: (i) providing cardio-protection and enhancing cardiac repair mechanisms; (ii) CPC biology; and (iii) influencing adult cardiomyocyte behavior.

## Introduction

Full cardiac regeneration is well-established in lower vertebrates including amphibians and the teleost fish; in the zebrafish heart, responsive reactivation of resident cardiomyocyte proliferation can occur following severe myocardial injury, such as apex resection, with almost total reconstitution of viable tissue over a transient scar (Lepilina et al., 2006; Kikuchi et al., 2010; Zhao et al., 2014; González-Rosa et al., 2017). On the contrary, the adult mammalian heart cannot withstand critical damage, as endowed with poor wound healing response when facing prolonged ischemia. Indeed, the heart responds to an ischemic injury – such as the occlusion of a coronary artery causing the death of a consistent amount of cardiomyocytes – by promptly triggering an inflammatory response with activation of myofibroblasts, which rapidly lay down a collagen-enriched scar tissue to compensate the cardiomyocyte loss. Such scar sustains mechanical strength and prevents ventricular rupture thereby avoiding sudden death. Despite this mechanism represents an emergency plan, it has detrimental consequences in the long term, including LV maladaptive remodeling with disruption of cardiac function by ventricular dilatation, scar thinning, and interstitial fibrosis, overall leading to HF (Pfeffer and Braunwald, 1990; Jameel and Zhang, 2009). Therefore, the emergency wound healing program can turn into defective repair, which is worsened by absence of efficient myocardial renewal and *de novo* cardiomyocyte restitution.

Restoration of both cardiac structure and function following injury, disease or aging represents the *holy grail* of modern medicine, yet current therapies can only delay progression of HF. In recent years, several preclinical and clinical efforts have focused on stem cell-based therapies, including different cell sources (i.e., BM- and adipose derived-MSC; fetal and perinatal progenitors, etc.) on the assumption that cells transplanted into the heart could give rise to new viable and functional cardiomyocytes and cardiovascular components via direct trans-differentiation. Despite initial high expectations, multiple independent lines of investigation have demonstrated that injected stem cells showed very low engraftment potential, poor survival and in most cases almost complete failure to acquire a mature cardiomyocyte phenotype, yet they contributed to improve cardiac function, mostly via local release of paracrine trophic factors. Indeed, accumulating evidence indicates that stem cells can prime the injured heart via paracrine effects, rather than undergoing *de novo* differentiation, as confirmed by the administration of their conditioned medium that contains all the secreted factors and showed equivalent beneficial results (Gnecchi et al., 2006). Thus, stem cell-derived paracrine modulation of cardiac tissue has recently emerged as a promising strategy for enhancing cardiac repair up to regeneration, with growing interest toward the functional profiling of the stem cell “*secretome*,” as the whole of growth factors, chemo-attractant molecules and EV produced by paracrine secretion.

In such scenario, much attention has been recently raised toward the strategic role of stem cell-EV as immunologically inert vehicles of regenerative activity and exerting pivotal function in cell-to-cell communication (Barile et al., 2014;Ibrahim et al., 2014). EV are a heterogeneous population of membrane-bound vesicles, including nano-sized exosomes (Ex) and micro-scaled shedding MV (Madonna et al., 2016; Sluijter et al., 2018). They contain proteins, bioactive lipids, mRNAs, and miRNAs molecules with relevant paracrine potential (Barile et al., 2014; Ibrahim et al., 2014). Notably, within the EV population, growing interest has been specifically dedicated to the exosome fraction; Ex represent a specific subpopulation of very small EV (ranging in size from 30 up to 150 nm), which are products of the endo-lysosomal pathway and have been described to be functional mediators of regenerative modulatory factors (Fierabracci et al., 2015; Radosinska and Bartekova, 2017). As such, EV and Ex have been increasingly analyzed in preclinical research, including the field of cancer and cardiac disease (Barile and Vassalli, 2017).

When addressing cardiac regeneration, two important aspects must be considered as well. First, recent independent studies have revealed that the heart does retain an endogenous regeneration capability, although very limited. Part of this potential is based on CPC activation. CPC are resident cells with stem/progenitor features mainly involved in cardiogenesis during embryonic development and participating in cardiac homeostasis and repair to some extent during adulthood. CPC represent a very heterogenous cell population as described so far, with putative tissue-specific cardiovascular and cardiomyogenic potential and significant paracrine activity, overall supporting cardiac function following injury (Bollini et al., 2011; Madonna et al., 2016). Their discovery has overturned the view of the heart as a non-regenerative organ; however, endogenous repair by CPC remains inefficient when facing severe pathological situations, unless they are activated by proper stimulation (Bollini et al., 2011; Laflamme and Murry, 2011). They represent a very appealing endogenous therapeutic target to sustain heart regeneration from within the cardiac tissue, therefore comprehensive characterization of CPC-EV and their biological activity has been lately gaining growing attention.

Likewise, the long-held view that mammalian cardiomyocytes are quiescent has been recently dismissed, although the number of mitotic cardiomyocytes drops precipitously within the first week of life. Thereafter, cardiomyocyte renewal rate remains at 0.5–1% per year in adult humans (Senyo et al., 2013). Despite being clearly insufficient to offset the loss of billion cardiomyocytes following myocardial infarct, a key question is whether this rate of proliferation can be therapeutically enhanced. Notably, it has been shown that in the neonatal mouse heart full regeneration following injury can be underpinned by active proliferation of existing mononuclear cardiomyocytes; unfortunately, this mechanism is transient, being lost after the first week of birth (P7), with transition from complete regeneration to scarring/fibrosis (Porrello et al., 2011).

Thus, the current cardiac regenerative dogma is based on synergistically optimizing cardiac repair, while also efficiently triggering both endogenous CPC reactivation and resident cardiomyocyte renewal, via paracrine effects. In this scenario, extensive comprehension of EV-mediated intercellular communication might offer meaningful insights for a future working strategy in translational medicine. In this review, we will critically examine the more relevant recent findings related to stem cell-EV biology for cardiac repair and regeneration, while also discussing the paracrine contribution of endogenous CPC and the emerging role of resident cardiomyocyte and the surrounding cardiac stromal cell secretory potential in modulating the tissue microenvironment via EV/Ex interaction.

## Stem Cell-EV for Future Cardiac Paracrine Therapy

### Stem Cell Biology 2.0: From the Genome to the Secretome

Within the (stem) cell-based therapy scenario, adult MSC have been the most investigated cell type in preclinical and clinical studies of cardiac repair, in either autologous as well as allogeneic setting. Despite their poor differentiating and engrafting potential, MSC are being considered a promising therapeutic source due to their remarkable cardioprotective, pro-angiogenic and anti-inflammatory paracrine potential. Indeed, in 2006 Gnecchi et al. published a pivotal study demonstrating that Akt-overexpressing MSC produced a cardioactive secretome by conditioning their culture medium, which exerted remarkable beneficial cardioprotective effect when administered to a preclinical rodent model of acute MI (Gnecchi et al., 2006). Since then, many independent studies confirmed the favorable paracrine effect on injured myocardium of different MSC sources. Transplanted MSC have been shown to instruct the host cardiac tissue by creating a responsive microenvironment, which is regulated by local concentration of secreted paracrine factors (Hodgkinson et al., 2016); hence, *in situ* modulation of host cellular responses is unlikely to be mediated by a single or by few factors, but rather by a more complex and synergistic combination of several paracrine agents, such as those conveyed by MSC-secreted EV. Indeed, stem cell-EV cargo can include a mixture of bio-active lipids, proteins and genetic information and has been increasingly scrutinized as therapeutic agent to enhance tissue repair (Lai et al., 2011; Chen et al., 2017).

### Shedding New Light on Stem Cell-EV

The shift in perspective from the stem cell genome to their secretome, with specific focus on the secreted EV, is transforming the idea of therapeutic application of stem cells in regenerative medicine. Indeed, by replacing cell transplantation with administration of secreted EV, many concerns and limits related to safety and feasibility could be mitigated. Exploiting stem cell-mediated effects via cell-free delivery of paracrine factors may result in a more feasible and clinically translational therapy. Mounting evidences support the working hypothesis of stem cell-EV as promising tool for therapeutic enhancement of cardiac repair mechanisms; several independent studies have reported that intra-myocardial injection of adult MSC-EV in rodent acute MI and I/R models markedly enhanced neovascularization, preserved cardiac function, reduced infarct size and counteracted pathological remodeling (Bian et al., 2014; Teng et al., 2015; Liu et al., 2017; Barile et al., 2018). Importantly some evidence suggests this may occur in a dose dependent fashion, as shown by Arslan et al. (2013). When injecting either 1, 4, or 16 μg/kg of exosomes intravenously in a mouse model of I/R, only the latter two were shown to have cardioprotective potential; this was further confirmed by using a 0.4 μg/ml MSC-Ex buffer in an *ex vivo* I/R injury setting (Arslan et al., 2013).

Therefore, in recent years growing interest has been addressed to the comprehensive characterization of the cardio-active paracrine profile of stem cell-secreted EV, in order to define an *advanced therapeutic medicinal product* (ATMP) for future cardiac regenerative medicine (Perrino et al., 2017; Sluijter et al., 2018). Consequently, much attention has been focused on the mechanisms of action dictated by their functional cargo. Despite being very small entities, stem cell-EV (and Ex in particular) represent relatively complex biological conveyors of inter-cellular communication via soluble factors as well as direct horizontal transfer of RNA molecules, mostly represented by miRNAs, as extensively reviewed in Hill et al. (2013); Lai et al. (2016), Bjørge et al. (2017), and Kim et al. (2017).

Indeed, an increasing number of investigations have been suggesting that stem cell-EV can mediate cardiac repair by delivering miRNA content to recipient cells within the heart, thus remarkably reprogramming target cell genetic information. Sahoo et al. (2011) were the first to report that human circulating CD34+ hematopoietic precursors release EV endowed with pro-angiogenic miR-130a and miR-126-3p. These EV had relevant potential in inducing neovascularization within the ischemic mouse tissue, as acting on VEGF-, ANG1-, ANG2-, and MMP9- signaling pathways (Sahoo et al., 2011; Mocharla et al., 2013; Mathiyalagan et al., 2017). Similarly, EV from human UC MSC have shown to contained various miRNAs crucial for cardiac and angiogenic cell differentiation of target cells, such as miR-199a-3p, miR-199a-5p, miR-23a-3p, miR-24a, miR-132-3p, miR-21-5p (Bobis-Wozowicz et al., 2016). Murine MSC have also been described to influence cardiac repair following MI in mice by miR-22 transfer via EV delivery, resulting in cardiomyocyte survival and inhibition of the profibrotic marker MeCP2 expression (Feng et al., 2014). Furthermore, the direct effect of EV/Ex cargo on cardio-protection was shown in a recent study by Luther et al. (2018) where an important contribution of miR21a-5p, present in MSC-Ex, was recognized both *in vitro* and *in vivo*. Importantly, by using Ex isolated from miR21a-knockout cells or by using miRNA mimics, they were able to conclude that the delivery of miRNA21a-5p into cardiomyocytes via exosomes and the downregulation of its target is at least partly responsible for apoptosis reduction *in vitro* and *in vivo*, with a smaller infarct size after I/R injury (Luther et al., 2018). Besides MSC, mouse embryonic stem cell (ESC)-EV, including Ex, significantly contributed to improve neovascularization, cardiomyocyte survival, and to limit fibrosis post infarction possibly acting via the miR-290-295 cluster (Khan et al., 2015). Despite the lack of a general consensus on the most cardio-active miRNA molecular signature within stem cell-EV/Ex, cumulative evidences, reinforced by comprehensive RNA sequencing, indicate the superior therapeutic effect of EV/Ex over MSC alone is due to their specific miRNA enrichment (Shao et al., 2017). Likewise, a recent study from our group indicated that human amniotic fluid stem cell-EV (hAFS-EV) are specifically enriched with miRNAs over proteins or other soluble factors; notably direct trafficking of miR-210 and miR-199a-3p from hAFS-EV to responder cells were suggested to drive pro-survival and proliferative effects in recipient human dermal fibroblast and murine myoblasts, which showed significant increase of such putative molecular candidates (Balbi et al., 2017). This may lay the foundations for further studies to pinpoint specific signaling pathway/molecular targets with relevant therapeutic application for cardiac repair and regeneration; indeed, these miRNAs have been described to exert remarkable effects on the myocardium *in vivo*, as potent drivers of local angiogenesis and cardiomyocyte renewal (Eulalio et al., 2012; Arif et al., 2017). As well, to instruct the cardiac tissue to enhance repair and counteract detrimental remodeling following injury, it is crucial to act within the very acute response phase; considering their prompt effect, timely modulation of specific signaling pathways by EV-mediated paracrine action may offer an interesting working strategy.

Nevertheless, additional functional mechanisms have been reported to elucidate stem cell-EV therapeutic effects. For example, Notch-pathway has been proposed as molecular candidate of paracrine intercellular communication, since Jagged 1-endowed MSC-EV induced robust and specific angiogenic response both *in vitro* and *ex vivo* by Matrigel plug assay (Gonzalez-King et al., 2017). As well, human ESC-derived MSC were shown to release Ex restoring the myocardium ATP production while decreasing oxidative stress via functional replenishment of glycolytic enzymes from their cargo into the injured myocardium in a preclinical mouse model of I/R injury (Arslan et al., 2013; Lai et al., 2013).

### Critical Aspects for Future Cardiac Paracrine Therapy

In the last few years a variety of stem cells have been scrutinized for cardiac repair, including peripheral blood-derived progenitor cells, BM- and adipose-derived MSC, fetal and perinatal stem and progenitor cells, embryonic and iPSC (as extensively reviewed in Abdelwahid et al., 2016). Given the promising cardioprotective and pro-angiogenic potential of stem cell-EV/Ex, many efforts have recently been focused on identifying the ideal cell source for scaling up the production of EV and Ex as advanced medicinal product for ischemic-related diseases.

**FIGURE 1:**
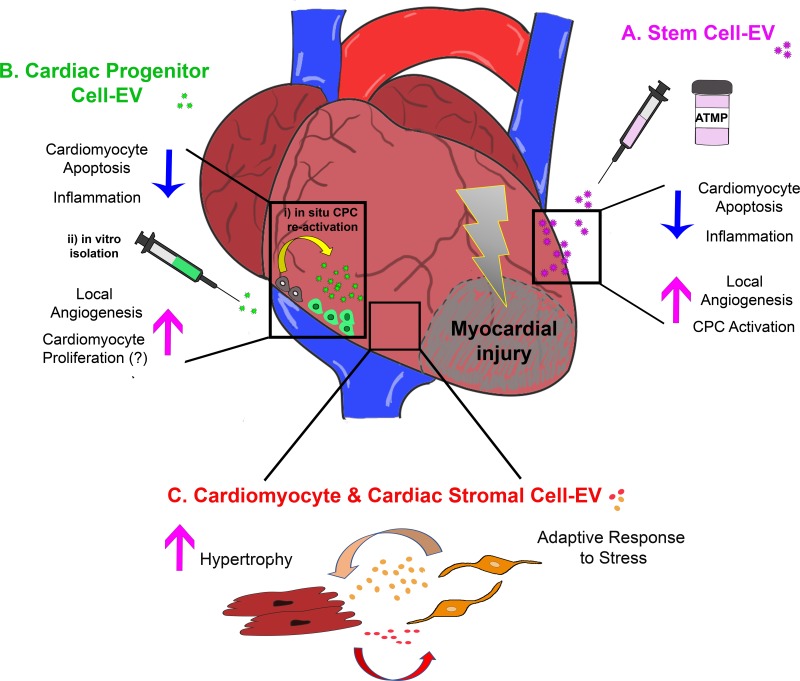
Schematic representation of the most relevant effects of extracellular vesicle-mediated intercellular communication within the injured myocardium as providing: **(A)** cardio-protection and cardiac repair by exogenous stem cell-EV/Ex; **(B)** paracrine modulation of the cardiac microenvironment by EV/Ex derived from CPC either activated *in situ* (i) or previously isolated and expanded *in vitro* (ii); and **(C)** fine tuning of adult resident cardiomyocyte and cardiac stromal cell behavior by their mutual EV/Ex modulatory exchange during adaptive response to stress. ATMP, advanced therapy medicinal product.

Cardiovascular disease patients need prompt therapeutic intervention and it would be ideal to have access to regenerative “off-the-shelf” products for simple administration; hence, stem cells might be used as a “drug store” to produce a highly efficient EV/Ex formulation to provide enhancement of cardiac repair (Figure 1). In this scenario, isolation feasibility and elevated self-renewal represents key aspects of the optimal stem cell source to be exploited for future paracrine therapy. Human adult progenitor cells, including MSC, can be isolated from a variety of post-natal tissues and obtained from discarded samples as clinical waste or leftover material during ordinary surgical or screening procedures (blood sampling, liposuction, BM transplantation, etc.). However, they can be affected by low yield and limited self-renewal potential, as they are often influenced by donor age (van Vliet et al., 2010). On the contrary, iPSC may overcome classical drawbacks of adult MSC by offering, in principle, unlimited production of stem and progenitor cells *in vitro*. Indeed, when comparing cardiac reparative effects of murine iPSC against their secreted EV, the latter showed superior paracrine potential in a preclinical model of I/R injury in mouse, thus representing an appealing therapeutic option by offering the benefits of iPSC therapy, but without the risk of tumorigenicity (Adamiak et al., 2017). Yet, iPSC technology can be challenging, costly and time consuming. The more recent characterization of fetal (with non-embryonic origin) and perinatal progenitor cells, have broadened the options. Fetal stem cells isolated either from AF (De Coppi et al., 2007) or villi (Poloni et al., 2008) as easily collected from leftover sample obtained during routine prenatal screening; perinatal stem cells can be isolated at birth from discarded extra-embryonic annexes, such as UC, including the WJ (Wang et al., 2004; Tauro et al., 2012), and placenta membranes (Magatti et al., 2016), thus representing an easily accessible source progenitors available in large amount and free from any ethical concern. Fetal and perinatal stem cells can offer specific advantages over adult MSC, since they are endowed with outstanding self-renewal and possibly higher paracrine potential than the adult ones, being developmentally more immature. Indeed, human UC-MSC-EV systemically injected into a preclinical rat model of MI sustained cardiac systolic function after 4 weeks, while offsetting fibrosis and cells apoptosis (Zhao et al., 2015). Specific interest has also been recently dedicated to the paracrine potential of human amniotic fluid-derived stem cell-EV, as described by few independent studies mediating therapeutic pro-survival, angiogenic, and anti-inflammatory effects (Balbi et al., 2017; Mellows et al., 2017; Sedrakyan et al., 2017; Beretti et al., 2018).

Stromal cell function and secretory potential can be critically determined by the microenvironment scenario. Notably, fine tuning of the microenvironment *in vitro* can significantly and quantitatively influence the cell secretome (Leuning et al., 2018). Therefore, *in vitro* cell-preconditioning can be adjusted in order to select the ideal cell culture conditions to prime stem cell-EV with optimal cardioprotective and pro-angiogenic potential for therapeutic relevance. For instance, short term hypoxic stimulation has been increasingly used to enrich MSC-EV and Ex with trophic paracrine factors. Delivery of these vesicles resulted in promotion of neovascularization and cardiac repair in preclinical rodent models of MI. The putative mechanisms of action have been reported to involve increased expression of nSMase2, which is critical for exosome biogenesis and EV-mediated transfer of miR-210 (Bian et al., 2014; Gonzalez-King et al., 2017; Zhu et al., 2017). Likewise, transgenic overexpression of specific cardio-active factors within stem cells has been employed to enhance the healing capacity of their secreted EV; overexpression of HIF-1α in human dental pulp-MSC led to secretion of enhanced Jagged 1-loaded exosomes that triggered the angiogenic differentiation of endothelial cells (Gonzalez-King et al., 2017). Moreover, Li et al. (2010) previously showed that rat (bone marrow) BM-MSC genetically modified to significantly increase GATA-4 expression, are endowed with noteworthy paracrine pro-survival and angiogenic potential in the ischemic environment. They then exploited the same working strategy to boost the cardioprotective paracrine effect of MSC-Ex in a preclinical rodent model of I/R injury; exosomal reprogramming of resident cardiomyocytes via the delivery of miR-19a targeting PTEN and BIM protein expression resulted in sustained activation of Akt and ERK signaling pathway, with significant recovery of cardiac function and decreased infarct size (Yu et al., 2015).

Stem cell-EV are being increasingly considered as biological enhancers of heart repair mechanisms (Madonna et al., 2016), yet little is known about their cardiac regenerative potential to trigger functional activation of endogenous CPC and responsive resident cardiomyocyte proliferation. Currently only a few studies have provided evidences of such critical effects. Mouse ESC-Ex have been demonstrated to prompt rodent resident c-KIT^+^ CPC survival, proliferation, and cardiac commitment several weeks after *in vivo* administration, with restitution of *de novo* cardiomyocytes in the ischemic heart, possibly via specific miR-294 transfer (Khan et al., 2015). Similarly, human iPSC-secreted shedding MV exerted *in vitro* proliferative and protective effects on human cardiac MSC possibly via direct transfer of miR-92b and elicited their cardiac and endothelial differentiation potential (Bobis-Wozowicz et al., 2015). Moreover, rat BM-MSC-Ex showed to prime c-KIT^+^ rat neonatal CPC by reprogramming their miRNA landscape, thus targeting a broad range of biological functions, from positive regulation of cell cycle, up to cell differentiation and response to hypoxia; such MSC-Ex preconditioning strategy also boosted CPC angiogenic ability and increased their *in vivo* functional potency after transplantation into the rat ischemic myocardium (Zhang et al., 2016). While such data is surely encouraging and very promising, further comprehensive studies are required to specifically assess stem cell-EV potential to sustain functional restoration of myocardial renewal via cardiomyocyte proliferation as well.

While standard cell therapy has been almost completely dismissed, high expectations have now been put into the therapeutic efficacy of stem cell-EV as paracrine facilitators of cardiac repair and regeneration. Indeed, they may represent an appealing alternative cell-free curative modality that could be clinically and translationally effective, safer, and cheaper. However, such a novel approach is still in its infancy as it requires extensive testing to validate EV safety and functional efficacy in the long term.

## A Closer Look: Endogenous Cardiac Progenitor Cell-EV for Repair and Regeneration

### Mending Broken Hearts From Within: The Biological Relevance of Cardiac Progenitor Cells

Cardiac progenitor cell represent a heterogenous population of cells scattered throughout the cardiac tissue, including atria, ventricles, and the epicardium (Bollini et al., 2011). They have been described expressing early cardiac development markers, thereby suggesting promising cardiovascular and cardiomyogenic potential which makes them particularly interesting as a therapeutic target for cardiac regeneration.

The first functional characterization of CPC came from a study by Beltrami et al. (2003), describing a population of resident progenitor cells found throughout the ventricular and atrial myocardium expressing markers that were up to that point associated with hematopoietic progenitor cells, including Stem cell antigen-1 (Sca-1) and the tyrosine kinase receptor c-KIT. Upon isolation, c-KIT^+^, Lin-, CD45- CPC were shown to differentiate into cardiac cell types and to contribute to repair of the injured myocardium when transplanted into a preclinical rat model of MI (Linke et al., 2005) by significantly decreasing scarring and fibrosis, and possibly by differentiating into cardiac cells. In the following years several other studies followed and focussed on putative different sub-populations of mammalian CPC (Gaetani et al., 2014). These were mainly based on the expression of well-recognized stem cells markers (i.e., c-KIT or Sca-1), by specific isolation and *in vitro* culture protocols, or by their developmental origin, as extensively reviewed in Bollini et al. (2011).

Within the human CPC scenario, two individual progenitor populations have been broadly investigated are the human c-KIT^+^ CPC and the so-called CS or CDC. Since these cells can be easily obtained from cardiac specimens obtained as clinical waste during routine cardiac surgery, their clinical translation was readily pursued. The promising results of c-KIT^+^ CPC transplantation in improving post-infarction left ventricular (LV) dysfunction in preclinical animal models, resulted in the SCIPIO-*Cardiac Stem Cell Infusion in Patients With Ischemic CardiOmyopathy* phase 1 clinical trial^1^. Despite initial reports suggesting that intracoronary infusion of autologous c-KIT^+^ CPC improved LV systolic function and reduced infarct size in patients with HF after MI (Bolli et al., 2011; Chugh et al., 2012) recently concern has been expressed over the integrity of previously published data (The Lancet Editors, 2014). CS, a heterogeneous population of cells able to spontaneously generate three-dimensional structures, have been described by Messina et al. (2004) to have stem-like, cardiovascular and cardiomyogenic properties (Smith et al., 2007) as well as regenerative influence on the damaged cardiac tissue (Johnston et al., 2009). Likewise, their promising profile led in 2009 to the CADUCEUS-*CArdiosphere-Derived aUtologous Stem CElls to Reverse ventricUlar dySfunction* clinical trial to test dose escalation safety and efficacy of intracoronary delivery in patients with ischemic LV dysfunction and MI. While no difference in LV ejection fraction was reported at 6 months follow-up, CPC-treated patients showed reduction in scar mass and improved regional contractility (Makkar et al., 2012).

Another human derived population that has been described, but has not yet been pursued for clinical application, is represented by Sca-1^+^ CPC. These cells have been shown to differentiate into beating cardiomyocytes and endothelial structures *in vitro* when stimulated with TGFβ or VEGF, respectively (Goumans et al., 2008). The transplantation of undifferentiated Sca-1^+^ CPC into the MI mouse heart resulted in enhanced cardiac function and expression of human cardiomyocyte markers at 12 weeks follow-up (Smits et al., 2009). However, in short-term follow-up, the positive effect on cardiac function was observed in absence of differentiation (Den Haan et al., 2012), suggesting a paracrine effect of the transplanted cells.

More recently, a population of CPC residing within the epicardium – the outer layer of the heart – has gained growing attention as a promising target for enhancing cardiac repair and regeneration (Moerkamp et al., 2016; Smits et al., 2018). During embryonic development there is a clear contribution of epicardial-derived cells (EPDC) to cardio-genesis. EPDC contribute to the smooth muscle component of the coronary vasculature, to interstitial fibroblasts and potentially to ventricular cardiomyocytes, although the latter is still under debate. Moreover, embryonic EPDC contribute soluble factors that stimulate the growth of the myocardium. In the adult heart, the epicardium is a quiescent layer, only to become activated after MI, as shown by an upregulation of embryonic markers, proliferation and migration (Zhou and Pu, 2011). Given the developmental ability of EPDC, and their partial activation post-injury, the epicardium and its derivatives are an intriguing endogenous cell type to pursue with respect to cardiac repair. Interestingly, Smart et al. (2011) showed that priming the epicardial response by systemic injection of the small cardio-active peptide thymosin β4 prior to infarction resulted in an increased activation and enhanced cardiac function, potentially accompanied by differentiation of EPDC into cardiomyocytes. EPDC can be isolated and cultured from different human cardiac samples including adult atrium samples and fetal cardiac specimens (van Tuyn et al., 2007; Moerkamp et al., 2016). When injected into to the infarcted mouse heart, EPDC where shown to positively affect cardiac function and stimulate local angiogenesis up to 6 weeks post-MI (Winter et al., 2007), but in the absence of the transplanted cells’ survival, thereby arguing for a paracrine contribution of EPDC to cardiac repair. This was further confirmed by studies where the conditioned medium of cultured mouse EPDC was shown to stimulate angiogenesis, and its injection into the heart resulted in reduced infarct size and enhanced cardiac function and local angiogenesis (Zhou et al., 2011).

### CPC Paracrine Potential via EV-Based Communication

Recent studies have emphasized that *ex vivo* cultured CPC show low engraftment and very limited differentiation potential within the injured myocardium (Winter et al., 2007; Smits et al., 2009; Feyen et al., 2017), while positive effects on cardiac function could have been recorded (Zwetsloot et al., 2016). Indeed, a very recent study based on an optimized genetic lineage trace has questioned the cardiomyogenic differentiation potential of the endogenous so-called cardiac “stem” cells or CSC, as showing that, opposite to what happens during embryonic cardiogenesis, in the adult heart *de novo* cardiomyocytes do not derive by putative resident cardiac stem cells (Li et al., 2018).

Thus, given that the biological profile of CPC is still quite controversial in terms of specific cardiovascular and cardiomyocyte commitment, there is now a general consensus on their substantial paracrine activity to exert cardioprotective effects, while increasing cardiac function. Hence, attention has moved to the paracrine profile of these cells with a particular interest in the functional characterization of CPC-EV, including Ex.

Indeed, several studies have highlighted the remarkable role of CPC-EV in enhancing cardiac repair mechanisms and promptly modulating acute inflammation, while sustaining long-term cardiac function. Ibrahim et al. (2014) recently showed that the EV secreted by CDC recapitulated the regenerative effects of the whole CDC secretome, including their pro-survival, proliferative and angiogenic effects on cardiac cells, both *in vitro* and *in vivo*. Similar beneficial results were also validated by an independent study from Barile et al. (2014) in which the cardioprotective effect of EV obtained from CPC derived from atrial appendage explants was tested against those derived from human dermal fibroblast. Indeed, human CPC-EV were shown to be the most cardio-active component of the progenitor cell paracrine secretome, as rodent infarcted hearts injected with EV from CPC, but not from control fibroblasts, prevented cardiomyocyte from apoptosis, enhanced neovascularization, and improved LV ejection fraction (Barile et al., 2014). Moreover, Tseliou et al. (2015) confirmed the therapeutic potential of cardiosphere-derived-EV in reprogramming fibroblast behavior and secretome, by making them less pro-fibrotic, more angiogenic and cardioprotective. Lately, human CDC-EV therapeutic feasibility has also been validated in a large animal preclinical model of MI in pigs, by intracoronary (IC) or open-chest intramyocardial (IM) delivery, after reperfusion in acute or chronic settings (Gallet et al., 2017; Nguyen et al., 2018). Local Ex delivery proved to be effective in decreasing fibrosis and adverse remodeling, while improving cardiac function after a single dose or follow up treatments in the long term; notably, the more clinically suitable IC administration did not produce equivalent beneficial results, suggesting that delivery route and timing are critical aspects for future cardiac paracrine therapy (Gallet et al., 2017).

CDC-Ex have been shown to horizontally transfer into target cells, resulting in critical decrease of scar mass and significant improvement of global cardiac function following MI. Notably, miR-146a was identified as particularly enriched within CDC-Ex and playing an important role in mediating some of the nanovesicle trophic effects, such as inhibiting pathological remodeling and increasing viable myocardium mass in a murine preclinical model of MI (Ibrahim et al., 2014). Yet, selective administration of miR-146a mimic reproduced only some -not all- the benefits obtained via CDC-Ex, suggesting that, while playing a significant role as part of their cargo, it cannot be considered as the individual molecular candidate to confer comprehensive therapeutic benefit to the injured heart. These results suggest a more cautious attitude toward the translation of EV/Ex biology into the clinical scenario and functional identification of discrete factors within their cargo as future pharmacological molecules with cardio-regenerative effects cannot be considered a straightforward approach yet.

Human CPC-EV have been also described as carrying cardio-active miRNAs including miR-210, miR-132, and miR-146a-3p, which affect the expression of Ephrin A3 and PTP1b in the responder cells, and subsequently lead to inhibition of cardiomyocyte apoptosis and enhancement of angiogenic differentiation by endothelial cells (Barile et al., 2014). Another relevant therapeutic miRNA enriched in CDC-EV is miRNA-181b (De Couto et al., 2017) as described to convey remarkable immunomodulatory effects, by decreasing CD68^+^ macrophages infiltrating in the heart after MI. Yet, miRNAs are not the only “usual suspects” driving EV- and Ex-derived regenerative effects. Recently, another class of non-coding RNA, the Y RNAs, has been reported to be plentiful in CPC-EV. Cambier et al. (2017) showed that a Y RNA fragment highly enriched in human CDC-EV can be actively transferred into macrophages, endowing them with cardioprotective potential and mediating strong anti-inflammatory and pro-survival effects in rodent hearts undergoing I/R injury (Cambier et al., 2017).

CPC-EV can directly deliver proteins to recipient cells as well. For instance, the pro-angiogenic effects of human Sca-1^+^ CPC-EV on endothelial cells *in vitro* and *in vivo* was shown to be regulated through high levels of EMMPRIN within these vesicles (Vrijsen et al., 2016). Notably, when exosome EMMPRIN was blocked via neutralizing antibodies, authors detected a significant reduction in EV-mediated angiogenic effects *in vitro*. Such result was further enhanced by shRNA-induced knock-down as EMMPRIN-deprived EV failed to stimulate network formation in a Matrigel tubule assay to the same extend as control untreated EV. Since enhancement of local angiogenesis is critical to optimize cardiac repair and improve functional recovery in the long term, this study provides interesting insights about exploiting specific EV molecular strength to drive effective pro-angiogenic responses within the damaged heart, such as vessel growth into the border zone and infarcted area. Similarly, a recent study compared the paracrine potential of human CPC-EV against the ones derived from bone marrow-derived mesenchymal stromal cell (BM-MSC), in an rodent model of MI (Barile et al., 2018). Results revealed that the superior cardioprotective role of CPC-EV could be related to a specific mechanism of action in the vesicle release of IGF-1 as mediated by PAPP-A, which was significantly enriched in the EV secreted by CPC over BM-MSC ones (Barile et al., 2018). The functional role of PAPP-A as master regulator of CPC-EV potential was further validated by transfecting cells with PAPP-A-specific siRNA; EV from PAPP-A-knockdown CPC did not inhibit cardiomyocyte apoptosis *in vitro*, nor counteracted LV dysfunction after permanent coronary ligation *in vivo* in rodents, hence highlighting the pivotal operative role of such protein on CPC-EV surface. Of interest, this study suggested a mechanism of action driving vesicle cardioprotective capacity other than the traditional view on EV mainly acting via intracellular delivery of their molecular cargo to target cells, hence providing new starting points for future strategies influencing cardiac repair and remodeling.

Several studies have addressed and comprehensively analyzed the paracrine potential of endogenous CPC-EV/Ex in optimizing cardiac repair by exerting beneficial cardio-protective, immunomodulatory, and angiogenic effects. Nevertheless, in order to define whether CPC-EV might represent an ideal future therapeutic candidate over exogenous stem cell-EV, further investigation should be provided on their cardio-active potential to restore and efficiently sustain myocardial renewal following injury. Indeed, so far very few studies have addressed such aspect, which is one of the most challenging for cardiac regenerative medicine. To the best of our knowledge, currently available data on CPC-EV potential to trigger cardiomyocyte proliferation is limited to *in vitro* analyses (Barile et al., 2014; Ibrahim et al., 2014).

Moreover, since CPC offer the unique advantage to mediate endogenous cardiac regeneration from within the cardiac tissue, a consensus on the most effective strategy to unlock their paracrine potential *in situ* is strongly needed. Indeed, adult CPC are usually quiescent or dormant and not efficiently responsive when facing injury or disease. Ideally, since they represent endogenous progenitors residing within the myocardial tissue, it would be advantageous to trigger their paracrine potential via EV release *in situ* by appropriate stimulation. Otherwise, CPC can be easily isolated from clinical waste specimen during routine cardiac surgery, *ex vivo* expanded and preconditioned with specific stimuli as for exogenous stem cells (Figure 1); however, being less immature and tissue-specific committed progenitors, such high level of processing can influence their behavior, secretome composition and yield.

## Resident Cardiovascular Cell Inter-Communication: the Cardiomyocyte-Fibroblast EV Axis

A whole body of experimental research indicates that cardiomyocytes and cardiac stromal cells, especially fibroblasts (Fib), influence one another via cell-to-cell contact and, to a larger extent, paracrine mediators (Howard and Baudino, 2014; Figure 1).

Several authors showed that co-culturing cardiac stromal cells and cardiomyocytes or incubating either cell population with the culture medium conditioned by the other one, results in significant structural and functional modifications. In most cases, it was demonstrated that cardiac Fib affect aspects of cardiomyocyte biology, such as hypertrophy (Cartledge et al., 2015), electrical activity (Pedrotty et al., 2009), contractility (LaFramboise et al., 2006), and viability (Shivakumar et al., 2008). Nonetheless, it was also found that other stromal cell types, such as endothelial cells, modulate cardiomyocytes in culture systems (Wang et al., 2014) as well as that cardiomyocytes may in turn regulate Fib and other non-cardiomyocyte populations (Cartledge et al., 2015; Garcia et al., 2016).

A second line of evidence consists of experiments with animal models, in which elimination of single cardiac Fib-secreted factors by genetic manipulation led to pathological ventricular remodeling in response to pressure overload (Pellieux et al., 2001; Sanada et al., 2007; Takeda et al., 2010). Overall, these studies have confirmed that the interaction between cardiomyocytes and non-cardiomyocytes described *in vitro* does occur also *in vivo*, and that any perturbation of this crosstalk invariably causes myocardial disease and HF. Interestingly, the same mediators may transmit signals from cardiomyocytes to stromal cells and vice versa. For instance, transgenic mice overexpressing the Notch ligand, Jagged1, on the surface of cardiomyocytes (which activates Notch signaling in contiguous cardiomyocytes and non-cardiomyocytes) display enhanced proliferation of CPC and cardiomyocytes in neonatal life, and reduced cardiomyocyte hypertrophy, blunted cardiac Fib expansion and fibrosis, and increased numbers of Sca-1- and Nkx2.5-positive CPC following pressure overload when adult (Nemir et al., 2014). On the other hand, genetic or pharmacological inhibition of Notch signaling in cardiac endothelial cells impairs the supply of fatty acids across the endothelium to cardiomyocytes, which thereby switch to maladaptive glucose-dependent energy metabolism; along with uncontrolled angiogenesis, this derangement promotes HF (Jabs et al., 2018).

Within this scenario of complex intercellular communications, a role for EV has been repeatedly proposed. In a seminal work, Thum et al. (2008) demonstrated that miR-21 stimulates cardiac Fib survival and activity, up to the point that silencing of miR-21 by a specific antagomir-21 significantly reduced LV fibrosis and dysfunction in mice subjected to pressure overload by transverse aortic constriction. Building on these findings, these and other authors subsequently showed that cardiac Fib secrete Ex enriched for miRNAs and miRNA passenger strands, despite these latter being predicted to be degraded during miRNA biogenesis (Bang et al., 2014). In particular, miR-21 passenger strand (miR-21^∗^) is highly expressed in Ex from cardiac Fib, is further increased by angiotensin II, and is implicated in hypertrophy of cardiomyocytes (Bang et al., 2014). Consistent with these observations, repression of miR21 by means of antagomir-21 and pharmacological blockade of miR-21^∗^ counteracted transverse aortic constriction- and angiotensin II-initiated LV hypertrophy, respectively (Thum et al., 2008; Bang et al., 2014). Other investigators subsequently confirmed that Ex are synthetized by cardiac Fib upon exposure to angiotensin II and cause cardiomyocyte hypertrophy in a paracrine manner, and that found that inhibition of Ex release attenuated angiotensin II-elicited cardiac hypertrophy (Lyu et al., 2015). Remarkably, cardiac Fib Ex were also shown to induce a cell-intrinsic renin-angiotensin system in cardiomyocytes (Lyu et al., 2015), and angiotensin II-primed cardiomyocytes were reported to induce miR21 signaling in cardiac Fib (Lorenzen et al., 2015), substantiating the concept that the crosstalk between cardiomyocytes and stromal cells is bidirectional and at multiple levels.

To add a further layer of complexity to the EV-mediated cell inter-communication within the myocardium, there is evidence that EV with potential paracrine effects are also released by cardiomyocytes, in spite of being these cells usually considered non-secretory. A pioneering study conducted in 2007 demonstrated the ability of cultured adult cardiomyocytes to secrete Ex containing HSP60, which is involved in regulation of cardiomyocytes apoptosis and inflammation and, therefore, may play a primary role in myocardial pathology (Tian et al., 2013). Expanding on these findings, it was subsequently observed that Ex secretion by cardiomyocytes is tightly regulated by the microenvironment and by ROS production and that cardiomyocytes-derived Ex have a peculiar protein content pattern, enriched for sarcomere and mitochondrial proteins, which is related to the stimuli driving EV formation (Malik et al., 2013). In fact, the effects of Ex from cardiomyocytes was different depending on the oxygen concentration to which cardiomyocytes were exposed: Ex released by cardiomyocytes in hypoxic condition displayed an increased cardioprotective activity compared to normoxic condition, with enhanced angiogenesis. This latter was at least in part due to higher levels of miR-222 and miR-143 (Ribeiro-Rodrigues et al., 2017). Other molecules highly present in cardiomyocyte EV are mRNAs, with 423 out of the 1520 mRNAs detected being connected in biological networks, in particular regarding energy metabolism (Waldenström et al., 2012).

A major limitation in investigating EV and Ex from cardiomyocytes is that it is not possible to set a human culture, and studies typically rely on various cell types – the murine cardiomyocyte HL-1 cell line, the rat cardiomyoblast H9c2 cell line, rat primary fetal cardiomyocytes and even adult rat cardiomyocytes (Gupta and Knowlton, 2007; Waldenström et al., 2012; Ribeiro-Rodrigues et al., 2017), none of which reliably reproduces the main features of human adult cardiomyocytes. To overcome this drawback, the use of cardiomyocytes obtained from iPSC may hold great promises (Denning et al., 2016).

## Open Questions

Cell-secreted vesicle biology is becoming a rapidly expanding field with dramatic impact on future cardiac regenerative medicine, given the noteworthy role of EV and Ex in driving heart repair and regeneration, while also regulating interaction among resident cardiomyocytes and surrounding stromal cells, as summarized in schematic Figure 1. Nonetheless, many critical aspects need further investigation in order to clearly define their clinical translational potential.

Extracellular vesicles represent a very heterogeneous population, being enriched with both Ex and MV. While several independent studies suggest Ex over MV as key mediators of biological modulatory effects (Barile and Vassalli, 2017; Mol et al., 2017), a general consensus on the most functionally cardio-active fraction between the two hasn’t been reached yet, since the comprehensive paracrine profile of Ex over MV is still undefined (Van Niel et al., 2018). Indeed, the structural heterogeneity of EV represents a challenge for our understanding of their biological functional role, as currently much remains unspecified regarding the detailed origin, regulatory pathways of secretion and cell targeting mechanisms. Likewise, tumorigenic Ex themselves have been lately described to be further fractioned into distinct subsets: large exosomes (ranging from 90 up to 120 nm), small exosomes (from 60 to 80 nm) and a third class of non-membranous nanoparticles defined as *exomeres* (about 35 nm). This additional sub-classification seems to be recapitulated by distinct proteomic, lipid, and nucleic acid content that influences organ biodistribution and pleiotropic effects related to cell metabolism, proliferation and secretion pathways (Zhang et al., 2018).

When considering clinical translation feasibility for future therapy, the overall reproducibility of the isolation methods as well as the efficiency of the product must be carefully considered. For example, it has yet to be determined whether endogenous CPC might be a preferential source over exogenous stem cells to harness cardio-active EV/Ex from. While CPC have to be either powerfully reactivated *in situ* with specific stimuli or harvested by invasive procedure and then expanded *in vitro* to exploit their paracrine potential, exogenous MSC can be obtained by a variety of clinical waste samples that may be much easily available. Likewise, iPSC may offer the advantage of being cultured in large quantities from a single isolation, providing a reliable background. Yet, given their ability to produce cardiac cell types, the CPC populations represent an interesting cell source to focus on, and may be especially efficient when cultured under conditions that resemble the injured myocardium (e.g., hypoxia or extracellular matrix composition).

Another relevant aspect influencing EV biology is related to their isolation protocol, from either cell-conditioned culture medium or biological fluids, including blood serum. Many different techniques have been reported and characterized: differential ultracentrifugation, immunoaffinity capture, ultrafiltration, size-exclusion chromatography, polymer-based precipitation up to state-of-the-art microfluidics (Théry et al., 2006; Chen et al., 2010; Merchant et al., 2010; Tauro et al., 2012; Jørgensen et al., 2013; Heinemann et al., 2014). Therefore, the ideal method for EV clinical translation and their scale-up standardization as future ATMP still represents a major challenge. Indeed, while most protocols aim at increase efficiency and efficacy of the isolation yield, many of them present relevant concerns about contamination of cell-derived molecules or do not offer reliable EV sub-fractioning into Ex versus MV from cell-conditioned medium as well as biological fluids (Baranyai et al., 2015; Sluijter et al., 2018). Recently, asymmetric flow field-flow fractioning has opened up a new promising scenario for the efficient isolation of distinct EV subpopulations, via highly reproducible, fast, simple, label-free technology (Zhang et al., 2018).

These methodological issues also represent a current major limitation to the possibility of measuring EV in the bloodstream for diagnosis of cardiac disease. In principle, increased circulating concentrations of EV containing myocardial-specific markers (proteins, lipids, and RNA) may allow the recognition of some cardiac disorders earlier and/or more accurately than by using conventional biomarkers, such as troponins and natriuretic peptides (Sluijter et al., 2018). In fact, seminal work has pinpointed EV content profiles selectively associated with acute coronary syndromes (de Hoog et al., 2013), HF (Wong et al., 2015), and chemotherapy-induced cardiomyopathy (Yarana et al., 2018). Nonetheless, the aforementioned technical aspects should not be overlooked and future prospective studies, designed *ad hoc*, are warranted to confirm the value of EV as biomarkers of cardiovascular disease in the clinical arena.

Extracellular vesicles participate in myocardial cell inter-communication and, in principle, novel therapies for LV hypertrophy and HF may target the cardiomyocyte-cardiac stromal cell paracrine axis or specific mediators that their EV may carry. Moreover, being EV also involved in physiological responses to stress, it will be extremely challenging to tailor innovative therapeutic approaches that inhibit detrimental effects, while preserving EV beneficial function in maintenance of cardiac homeostasis.

As several studies have emphasized the direct effect on EV cargo on their target cells to influence for instance angiogenesis (Vrijsen et al., 2016) or cardio-protection (Luther et al., 2018), establishing whether the combination of discrete paracrine factors (either distinct soluble factors or nucleic acid information) identified within the vesicle cargo can completely recapitulate EV cardioprotective and/or regenerative effects can offer critical information for future pharmacological applications. As well, the most efficient and less invasive administration protocol for putative EV-based cardiac paracrine therapy has to be characterized yet; indeed, while single administration during angioplasty procedure may be clinically feasible, maintenance of therapeutic levels in the long term might require follow-up treatments which should be delivered to the myocardial tissue as exclusively as possible. Moreover, dose-response effects need to be comprehensively investigated; currently information on this specific aspect is quite limited and general consensus on the most appropriate method to measure the EV dose to be employed has not been reached yet. Indeed, in most studies the dose of EV administered *in vitro* and/or *in vivo* has been indicated by number of EV particles/μl (Ibrahim et al., 2014; Gallet et al., 2017; Barile et al., 2018) or by μg of EV total protein (Arslan et al., 2013; Barile et al., 2014; Balbi et al., 2017), or as the amount of EV obtain by a specific number of producing cells (Sahoo et al., 2011). Likewise, pharmacokinetics and pharmacodynamics of EV-based formulations need to be comprehensively investigated in order to avoid any potential adverse side effects of therapeutic dose, while ensuring efficacy.

Overall, EV biology surely represents a fascinating and promising field to be therapeutically exploited for future cardiac repair and regeneration strategies; yet our understandings of EV basic mechanisms of action within the myocardial tissue, from their biogenesis to cell targeting and specific delivery of the informative content, need to be significantly improved.

## Author Contributions

SB contributed to conception and design; manuscript writing and revision; critical discussion of stem cell-EV characterization and potential for cardiac repair and regeneration; and final approval of manuscript. AS contributed to manuscript writing and revision; analytical discussion of CPC biology; and final approval of manuscript. CB contributed to manuscript writing and critical discussion on CPC-EV/Ex; and final approval of manuscript. EL contributed to manuscript writing and critical discussion on cardiac stromal cell paracrine potential; and final approval of manuscript. PA contributed to manuscript writing and revision, analytical discussion of secretory potential of cardiomyocyte and cardiac stromal cells; and final approval of manuscript.

## Conflict of Interest Statement

The authors declare that the research was conducted in the absence of any commercial or financial relationships that could be construed as a potential conflict of interest.

## Footnotes

1 https://clinicaltrials.gov/ct2/show/NCT00474461

## Abbreviations

AF: amniotic fluid
ANG1: angiopoietin 1
ANG2: angiopoietin 2
ATMP: advanced therapy medicinal product
BM: bone marrow
CDC: cardiosphere-derived progenitor cells
CPC: cardiac progenitor cell
CS: cardiospheres
EMMPRIN: extracellular matrix metalloproteinase inducer
EPDC: epicardium-derived progenitor cells
ERK: extracellular signal–regulated kinase
ESC: embryonic stem cells
EV: extracellular vesicles
Ex: exosomes
Fib: fibroblasts
HF: heart failure
HIF: hypoxia inducible factor
HSP60: heat shock protein 60
I/R: ischemia/reperfusion
IC: intra-coronary
IM: intra-myocardial
iPSC: induced pluripotent stem cells
Lin-: lineage-negative
LV: left ventricle
MeCP2: Methyl-CpG binding protein 2
MI: myocardial infarction
miRNA: microRNA
MMP9: matrix metallopeptidase 9
mRNA: messenger RNA
MSC: mesenchymal stromal cells
MV: microvesicles
nSMase2: neutral sphingomyelinase 2
PAPP-A: pregnancy-associated plasma protein-A
ROS: reactive oxygen species
UC: umbilical cord
VEGF: vascular endothelial growth factor
WJ: Wharton’s jelly

## References

[B1] Abdelwahid E. Kalvelyte A. Stulpinas A. de Carvalho K. A. T. Guarita-Souza L. C. Foldes G. (2016). Stem cell death and survival in heart regeneration and repair. *Apoptosis* 21 252–68. 10.1007/s10495-015-1203-4 26687129 PMC5200890

[B2] Adamiak M. Cheng G. Bobis-Wozowicz S. Zhao L. Kedracka-Krok S. Samanta A. (2017). Induced pluripotent stem cell (iPSC)-derived extracellular vesicles are safer and more effective for cardiac repair than iPSCs. *Circ. Res.* 122:CIRCRESAHA.117.311769. 10.1161/CIRCRESAHA.117.311769 29118058 PMC5775034

[B3] Arif M. Pandey R. Alam P. Jiang S. Sadayappan S. Paul A. (2017). MicroRNA-210-mediated proliferation, survival, and angiogenesis promote cardiac repair post myocardial infarction in rodents. *J. Mol. Med. (Berl.)* 95 1369–1385. 10.1007/s00109-017-1591-8 28948298 PMC5941944

[B4] Arslan F. Lai R. C. Smeets M. B. Akeroyd L. Choo A. Aguor E. N. E. (2013). Mesenchymal stem cell-derived exosomes increase ATP levels, decrease oxidative stress and activate PI3K/Akt pathway to enhance myocardial viability and prevent adverse remodeling after myocardial ischemia/reperfusion injury. *Stem Cell Res.* 10 301–312. 10.1016/j.scr.2013.01.002 23399448

[B5] Balbi C. Piccoli M. Barile L. Papait A. Armirotti A. Principi E. (2017). First characterization of human amniotic fluid stem cell extracellular vesicles as a powerful paracrine tool endowed with regenerative potential. *Stem Cells Transl. Med.* 6 1340–1355. 10.1002/sctm.16-0297 28271621 PMC5442724

[B6] Bang C. Batkai S. Dangwal S. Gupta S. K. Foinquinos A. Holzmann A. (2014). Cardiac fibroblast-derived microRNA passenger strand-enriched exosomes mediate cardiomyocyte hypertrophy. *J. Clin. Invest.* 124 2136–2146. 10.1172/JCI70577 24743145 PMC4001534

[B7] Baranyai T. Herczeg K. Onódi Z. Voszka I. Módos K. Marton N. (2015). Isolation of exosomes from blood plasma: qualitative and quantitative comparison of ultracentrifugation and size exclusion chromatography methods. *PLoS One* 10:e0145686. 10.1371/journal.pone.0145686 26690353 PMC4686892

[B8] Barile L. Cervio E. Lionetti V. Milano G. Ciullo A. Biemmi V. (2018). Cardioprotection by cardiac progenitor cell-secreted exosomes: role of pregnancy-associated plasma protein-A. *Cardiovasc. Res.* 114 992–1005. 10.1093/cvr/cvy055 29518183

[B9] Barile L. Lionetti V. Cervio E. Matteucci M. Gherghiceanu M. Popescu L. M. (2014). Extracellular vesicles from human cardiac progenitor cells inhibit cardiomyocyte apoptosis and improve cardiac function after myocardial infarction. *Cardiovasc. Res.* 103 530–541. 10.1093/cvr/cvu167 25016614

[B10] Barile L. Vassalli G. (2017). Exosomes: therapy delivery tools and biomarkers of diseases. *Pharmacol. Ther.* 174 63–78. 10.1016/j.pharmthera.2017.02.020 28202367

[B11] Beltrami A. P. Barlucchi L. Torella D. Baker M. Limana F. Chimenti S. (2003). Adult cardiac stem cells are multipotent and support myocardial regeneration. *Cell* 114 763–776. 10.1016/S0092-8674(03)00687-1 14505575

[B12] Beretti F. Zavatti M. Casciaro F. Comitini G. Franchi F. Barbieri V. (2018). Amniotic fluid stem cell exosomes: therapeutic perspective. *Biofactors* 44 158–167. 10.1002/biof.1407 29341292

[B13] Bian S. Zhang L. Duan L. Wang X. Ying M. Yu H. (2014). Extracellular vesicles derived from human bone marrow mesenchymal stem cells promote angiogenesis in a rat myocardial infarction model. *J. Mol. Med.* 92 387–397. 10.1007/s00109-013-1110-5 24337504

[B14] Bjørge I. M. Kim S. Y. Mano J. F. Kalionis B. Chrzanowski W. (2017). Extracellular vesicles, exosomes and shedding vesicles in regenerative medicine – A new paradigm for tissue repair. *Biomater. Sci.* 6 60–78. 10.1039/C7BM00479F 29184934

[B15] Bobis-Wozowicz S. Kmiotek K. Kania K. Karnas E. Labedz-Maslowska A. Sekula M. (2016). Diverse impact of xeno-free conditions on biological and regenerative properties of hUC-MSCs and their extracellular vesicles. *J. Mol. Med.* 95 205–220. 10.1007/s00109-016-1471-7 27638341 PMC5239805

[B16] Bobis-Wozowicz S. Kmiotek K. Sekula M. Kedracka-Krok S. Kamycka E. Adamiak M. (2015). Human induced pluripotent stem cell-derived microvesicles transmit RNAs and proteins to recipient mature heart cells modulating cell fate and behavior. *Stem Cells* 33 2748–2761. 10.1002/stem.2078 26031404

[B17] Bolli R. Chugh A. R. D’Amario D. Loughran J. H. Stoddard M. F. Ikram S. (2011). Cardiac stem cells in patients with ischaemic cardiomyopathy (SCIPIO): Initial results of a randomised phase 1 trial. *Lancet* 378 1847–1857. 10.1016/S0140-6736(11)61590-0 22088800 PMC3614010

[B18] Bollini S. Smart N. Riley P. R. (2011). Resident cardiac progenitor cells: at the heart of regeneration. *J. Mol. Cell. Cardiol.* 50 296–303. 10.1016/j.yjmcc.2010.07.006 20643135

[B19] Cambier L. de Couto G. Ibrahim A. Echavez A. K. Valle J. Liu W. (2017). Y RNA fragment in extracellular vesicles confers cardioprotection via modulation of IL-10 expression and secretion. *EMBO Mol. Med.* 9 337–352. 10.15252/emmm.201606924 28167565 PMC5331234

[B20] Cartledge J. E. Kane C. Dias P. Tesfom M. Clarke L. Mckee B. (2015). Functional crosstalk between cardiac fibroblasts and adult cardiomyocytes by soluble mediators. *Cardiovasc. Res.* 105 260–270. 10.1093/cvr/cvu264 25560320

[B21] Chen B. Li Q. Zhao B. Wang Y. (2017). Stem cell-derived extracellular vesicles as a novel potential therapeutic tool for tissue repair. *Stem Cells Transl. Med.* 6 1753–1758. 10.1002/sctm.16-0477 28653443 PMC5689748

[B22] Chen C. Skog J. Hsu C.-H. Lessard R. T. Balaj L. Wurdinger T. (2010). Microfluidic isolation and transcriptome analysis of serum microvesicles. *Lab Chip* 10 505–511. 10.1039/B916199F 20126692 PMC3136803

[B23] Chugh A. R. Beache G. M. Loughran J. H. Mewton N. Elmore J. B. Kajstura J. (2012). Administration of cardiac stem cells in patients with ischemic cardiomyopathy: the SCIPIO trial: surgical aspects and interim analysis of myocardial function and viability by magnetic resonance. *Circulation* 126 S54–S64. 10.1161/CIRCULATIONAHA.112.092627 22965994 PMC3448934

[B24] De Coppi P. Bartsch G. Siddiqui M. M. Xu T. Santos C. C. Perin L. (2007). Isolation of amniotic stem cell lines with potential for therapy. *Nat. Biotechnol.* 25 100–106. 10.1038/nbt1274 17206138

[B25] De Couto G. Gallet R. Cambier L. Jaghatspanyan E. Makkar N. Dawkins J. F. (2017). Exosomal MicroRNA transfer into macrophages mediates cellular postconditioning. *Circulation* 136 200–214. 10.1161/CIRCULATIONAHA.116.024590 28411247 PMC5505791

[B26] de Hoog V. C. Timmers L. Schoneveld A. H. Wang J.-W. van de Weg S. M. Sze S. K. (2013). Serum extracellular vesicle protein levels are associated with acute coronary syndrome. *Eur. Hear. J. Acute Cardiovasc. Care* 2 53–60. 10.1177/2048872612471212 24062934 PMC3760575

[B27] Den Haan M. C. Grauss R. W. Smits A. M. Winter E. M. Van Tuyn J. Pijnappels D. A. (2012). Cardiomyogenic differentiation-independent improvement of cardiac function by human cardiomyocyte progenitor cell injection in ischaemic mouse hearts. *J. Cell. Mol. Med.* 16 1508–1521. 10.1111/j.1582-4934.2011.01468.x 22003890 PMC3823219

[B28] Denning C. Borgdorff V. Crutchley J. Firth K. S. A. George V. Kalra S. (2016). Cardiomyocytes from human pluripotent stem cells: from laboratory curiosity to industrial biomedical platform. *Biochim. Biophys. Acta – Mol. Cell Res.* 1863 1728–1748. 10.1016/j.bbamcr.2015.10.014 26524115 PMC5221745

[B29] Eulalio A. Mano M. Dal Ferro M. Zentilin L. Sinagra G. Zacchigna S. (2012). Functional screening identifies miRNAs inducing cardiac regeneration. *Nature* 492 376–381. 10.1038/nature11739 23222520

[B30] Feng Y. Huang W. Wani M. Yu X. Ashraf M. (2014). Ischemic preconditioning potentiates the protective effect of stem cells through secretion of exosomes by targeting Mecp2 via miR-22. *PLoS One* 9:e88685. 10.1371/journal.pone.0088685 24558412 PMC3928277

[B31] Feyen D. A. M. Van Den Hoogen P. Van Laake L. W. Van Eeuwijk E. C. M. Hoefer I. Pasterkamp G. (2017). Intramyocardial stem cell injection: Go(ne) with the flow Frederieke van den Akker1. *Eur. Heart J.* 38 184–186. 10.1093/eurheartj/ehw056 28158468

[B32] Fierabracci A. Del Fattore A. Luciano R. Muraca M. Teti A. Muraca M. (2015). Recent advances in mesenchymal stem cell immunomodulation: the role of microvesicles. *Cell Transplant.* 24 133–149. 10.3727/096368913X675728 24268069

[B33] Gaetani R. Feyen D. A. M. Doevendans P. A. Gremmels H. Forte E. Fledderus J. O. (2014). Different types of cultured human adult cardiac progenitor cells have a high degree of transcriptome similarity. *J. Cell. Mol. Med.* 18 2147–2151. 10.1111/jcmm.12458 25311343 PMC4224548

[B34] Gallet R. Dawkins J. Valle J. Simsolo E. de Couto G. Middleton R. (2017). Exosomes secreted by cardiosphere-derived cells reduce scarring, attenuate adverse remodelling, and improve function in acute and chronic porcine myocardial infarction. *Eur. Heart J.* 38 201–211. 10.1093/eurheartj/ehw240 28158410 PMC5837390

[B35] Garcia N. A. Moncayo-Arlandi J. Sepulveda P. Diez-Juan A. (2016). Cardiomyocyte exosomes regulate glycolytic flux in endothelium by direct transfer of GLUT transporters and glycolytic enzymes. *Cardiovasc. Res.* 109 397–408. 10.1093/cvr/cvv260 26609058

[B36] Gnecchi M. He H. Noiseux N. Liang O. D. Zhang L. Morello F. (2006). Evidence supporting paracrine hypothesis for Akt-modified mesenchymal stem cell-mediated cardiac protection and functional improvement. *FASEB J.* 20 661–669. 10.1096/fj.05-5211com 16581974

[B37] Gonzalez-King H. García N. A. Ontoria-Oviedo I. Ciria M. Montero J. A. Sepúlveda P. (2017). Hypoxia inducible factor-1α potentiates jagged 1-mediated angiogenesis by mesenchymal stem cell-derived exosomes. *Stem Cells* 35 1747–1759. 10.1002/stem.2618 28376567

[B38] González-Rosa J. M. Burns C. E. Burns C. G. (2017). Zebrafish heart regeneration: 15 years of discoveries. *Regeneration* 4 105–123. 10.1002/reg2.83 28979788 PMC5617908

[B39] Goumans M. J. de Boer T. P. Smits A. M. van Laake L. W. van Vliet P. Metz C. H. G. (2008). TGF-β1 induces efficient differentiation of human cardiomyocyte progenitor cells into functional cardiomyocytes in vitro. *Stem Cell Res.* 1 138–149. 10.1016/j.scr.2008.02.003 19383394

[B40] Gupta S. Knowlton A. A. (2007). HSP60 trafficking in adult cardiac myocytes: role of the exosomal pathway. *Am. J. Physiol. Heart Circ. Physiol.* 292 H3052–H3056. 10.1152/ajpheart.01355.2006 17307989

[B41] Heinemann M. L. Ilmer M. Silva L. P. Hawke D. H. Recio A. Vorontsova M. A. (2014). Benchtop isolation and characterization of functional exosomes by sequential filtration. *J. Chromatogr. A* 1371 125–135. 10.1016/j.chroma.2014.10.026 25458527

[B42] Hill A. F. Pegtel D. M. Lambertz U. Leonardi T. O’Driscoll L. Pluchino S. (2013). ISEV position paper: extracellular vesicle RNA analysis and bioinformatics. *J. Extracell. Vesicles* 2:22859. 10.3402/jev.v2i0.22859 24376909 PMC3873759

[B43] Hodgkinson C. P. Bareja A. Gomez J. A. Dzau V. J. (2016). Emerging concepts in paracrine mechanisms in regenerative cardiovascular medicine and biology. *Circ. Res.* 118 95–107. 10.1161/CIRCRESAHA.115.305373 26837742 PMC4874329

[B44] Howard C. M. Baudino T. A. (2014). Dynamic cell-cell and cell-ECM interactions in the heart. *J. Mol. Cell. Cardiol.* 70 19–26. 10.1016/j.yjmcc.2013.10.006 24140801

[B45] Ibrahim A. G. Cheng K. Marbán E. (2014). Exosomes as critical agents of cardiac regeneration triggered by cell therapy. *Stem Cell Rep.* 2 606–619. 10.1016/j.stemcr.2014.04.006 24936449 PMC4050492

[B46] Jabs M. Rose A. J. Lehmann L. H. Taylor J. Moll I. Sijmonsma T. P. (2018). Inhibition of endothelial notch signaling impairs fatty acid transport and leads to metabolic and vascular remodeling of the adult heart. *Circulation* 137 2592–2608. 10.1161/CIRCULATIONAHA.117.029733 29353241

[B47] Jameel M. N. Zhang J. (2009). Heart failure management: the present and the future. *Antioxid. Redox Signal.* 11 1989–2010. 10.1089/ars.2009.2488 19203220 PMC2810134

[B48] Johnston P. V. Sasano T. Mills K. Evers R. Lee S.-T. Smith R. R. (2009). Engraftment, differentiation, and functional benefits of autologous cardiosphere-derived cells in porcine ischemic cardiomyopathy. *Circulation* 120 1075–1083. 10.1161/CIRCULATIONAHA.108.816058 19738142 PMC2848167

[B49] Jørgensen M. Bæk R. Pedersen S. Søndergaard E. K. L. Kristensen S. R. Varming K. (2013). Extracellular vesicle (EV) array: microarray capturing of exosomes and other extracellular vesicles for multiplexed phenotyping. *J. Extracell. Vesicles* 2:20920. 10.3402/jev.v2i0.20920 24009888 PMC3760630

[B50] Khan M. Nickoloff E. Abramova T. Johnson J. Verma S. K. Krishnamurthy P. (2015). Embryonic stem cell-derived exosomes promote endogenous repair mechanisms and enhance cardiac function following myocardial infarction. *Circ. Res.* 117 52–64. 10.1161/CIRCRESAHA.117.305990 25904597 PMC4482130

[B51] Kikuchi K. Holdway J. E. Werdich A. A. Anderson R. M. Fang Y. Egnaczyk G. F. (2010). Primary contribution to zebrafish heart regeneration by gata4+ cardiomyocytes. *Nature* 464 601–605. 10.1038/nature08804 20336144 PMC3040215

[B52] Kim K. M. Abdelmohsen K. Mustapic M. Kapogiannis D. Gorospe M. (2017). RNA in extracellular vesicles. *Wiley Interdiscip. Rev. RNA* 8:e1413. 10.1002/wrna.1413 28130830 PMC5474163

[B53] Laflamme M. A. Murry C. E. (2011). Heart regeneration. *Nature* 473 326–335. 10.1038/nature10147 21593865 PMC4091722

[B54] LaFramboise W. A. Scalise D. Stoodley P. Graner S. R. Guthrie R. D. Magovern J. A. (2006). Cardiac fibroblasts influence cardiomyocyte phenotype in vitro. *AJP Cell Physiol.* 292 C1799–C1808. 10.1152/ajpcell.00166.2006 17229813

[B55] Lai R. C. Chen T. S. Lim S. K. (2011). Mesenchymal stem cell exosome: a novel stem cell-based therapy for cardiovascular disease. *Regen. Med.* 6 481–492. 10.2217/rme.11.35 21749206

[B56] Lai R. C. Tan S. S. Yeo R. W. Y. Choo A. B. H. Reiner A. T. Su Y. (2016). MSC secretes at least 3 EV types each with a unique permutation of membrane lipid, protein and RNA. *J. Extracell. Vesicles* 5:29828. 10.3402/jev.v5.29828 26928672 PMC4770866

[B57] Lai R. C. Yeo R. W. Y. Tan K. H. Lim S. K. (2013). Mesenchymal stem cell exosome ameliorates reperfusion injury through proteomic complementation. *Regen. Med.* 8 197–209. 10.2217/rme.13.4 23477399

[B58] Lepilina A. Coon A. N. Kikuchi K. Holdway J. E. Roberts R. W. Burns C. G. (2006). A dynamic epicardial injury response supports progenitor cell activity during zebrafish heart regeneration. *Cell* 127 607–619. 10.1016/j.cell.2006.08.052 17081981

[B59] Leuning D. G. Beijer N. R. M. Fossé N. A. Vermeulen S. Lievers E. Van Kooten C. (2018). The cytokine secretion profile of mesenchymal stromal cells is determined by surface structure of the microenvironment. *Sci. Rep.* 8 1–9. 10.1038/s41598-018-25700-5 29769543 PMC5956003

[B60] Li H. Zuo S. He Z. Yang Y. Pasha Z. Wang Y. (2010). Paracrine factors released by GATA-4 overexpressed mesenchymal stem cells increase angiogenesis and cell survival. *AJP Hear. Circ. Physiol.* 299 H1772–H1781. 10.1152/ajpheart.00557.2010 20870802 PMC3006287

[B61] Li Y. He L. Huang X. Issa Bhaloo S. Zhao H. Zhang S. (2018). Genetic lineage tracing of non-myocyte population by dual recombinases. *Circulation* 10.1161/CIRCULATIONAHA.118.034250 [Epub ahead of print]. 29700121

[B62] Linke A. Muller P. Nurzynska D. Casarsa C. Torella D. Nascimbene A. (2005). Stem cells in the dog heart are self-renewing, clonogenic, and multipotent and regenerate infarcted myocardium, improving cardiac function. *Proc. Natl. Acad. Sci. U.S.A.* 102 8966–8971. 10.1073/pnas.0502678102 15951423 PMC1157041

[B63] Liu L. Jin X. Hu C. F. Li R. Zhou Z. Shen C. X. (2017). Exosomes derived from mesenchymal stem cells rescue myocardial ischaemia/reperfusion injury by inducing cardiomyocyte autophagy via AMPK and Akt Pathways. *Cell. Physiol. Biochem.* 43 52–68. 10.1159/000480317 28848091

[B64] Lorenzen J. M. Schauerte C. Hübner A. Kölling M. Martino F. Scherf K. (2015). Osteopontin is indispensible for AP1-mediated angiotensin II-related miR-21 transcription during cardiac fibrosis. *Eur. Heart J.* 36 2184–2196. 10.1093/eurheartj/ehv109 25898844 PMC4543785

[B65] Luther K. M. Haar L. McGuinness M. Wang Y. Lynch Iv T. L. (2018). Exosomal miR-21a-5p mediates cardioprotection by mesenchymal stem cells. *J. Mol. Cell. Cardiol.* 119 125–137. 10.1016/j.yjmcc.2018.04.012 29698635

[B66] Lyu L. Wang H. Li B. Qin Q. Qi L. Nagarkatti M. (2015). A critical role of cardiac fibroblast-derived exosomes in activating renin angiotensin system in cardiomyocytes. *J. Mol. Cell. Cardiol.* 89 268–279. 10.1016/j.yjmcc.2015.10.022 26497614 PMC4988239

[B67] Madonna R. Van Laake L. W. Davidson S. M. Engel F. B. Hausenloy D. J. Lecour S. (2016). Position paper of the european society of cardiology working group cellular biology of the heart: cell-based therapies for myocardial repair and regeneration in ischemic heart disease and heart failure. *Eur. Heart J.* 37 1789–1798. 10.1093/eurheartj/ehw113 27055812 PMC4912026

[B68] Magatti M. Pianta S. Silini A. Parolini O. (2016). Isolation, culture, and phenotypic characterization of mesenchymal stromal cells from the amniotic membrane of the human term placenta. *Methods Mol. Biol. (Clifton, N.J.)* 1416 233–244. 10.1007/978-1-4939-3584-0_13 27236675

[B69] Makkar R. R. Smith R. R. Cheng K. Malliaras K. Thomson L. E. J. Berman D. (2012). Intracoronary cardiosphere-derived cells for heart regeneration after myocardial infarction (CADUCEUS): a prospective, randomised phase 1 trial. *Lancet (London, England)* 379 895–904. 10.1016/S0140-6736(12)60195-0 22336189 PMC4326004

[B70] Malik Z. A. Kott K. S. Poe A. J. Kuo T. Chen L. Ferrara K. W. (2013). Cardiac myocyte exosomes: stability, HSP60, and proteomics. *Am. J. Physiol. Heart Circ. Physiol.* 304 H954–H965. 10.1152/ajpheart.00835.2012 23376832 PMC3625894

[B71] Mathiyalagan P. Liang Y. Kim D. Misener S. Thorne T. Kamide C. E. (2017). Angiogenic mechanisms of human CD34 + stem cell exosomes in the repair of ischemic hindlimb. *Circ. Res.* 120 1466–1476. 10.1161/CIRCRESAHA.116.310557 28298297 PMC5420547

[B72] Mellows B. Mitchell R. Antonioli M. Kretz O. Chambers D. Zeuner M.-T. (2017). Protein and molecular characterization of a clinically compliant amniotic fluid stem cell-derived extracellular vesicle fraction capable of accelerating muscle regeneration through enhancement of angiogenesis. *Stem Cells Dev.* 26 1316–1333. 10.1089/scd.2017.0089 28679310

[B73] Merchant M. L. Powell D. W. Wilkey D. W. Cummins T. D. Deegens J. K. Rood I. M. (2010). Microfiltration isolation of human urinary exosomes for characterization by MS. *Proteomics – Clin. Appl.* 4 84–96. 10.1002/prca.200800093 21137018

[B74] Messina E. Angelis L. De Frati G. Morrone S. Chimenti S. Fiordaliso F. (2004). Isolation and expansion of adult cardiac stem cells from human and murine heart. *Circ. Res.* 95 911–921. 10.1161/01.RES.0000147315.71699.51 15472116

[B75] Mocharla P. Briand S. Giannotti G. Dörries C. Jakob P. Paneni F. (2013). AngiomiR-126 expression and secretion from circulating CD34 + and CD14 + PBMCs: role for proangiogenic effects and alterations in type 2 diabetics. *Blood* 121 226–236. 10.1182/blood-2012-01-407106 23144172

[B76] Moerkamp A. T. Lodder K. van Herwaarden T. Dronkers E. Dingenouts C. K. E. Tengström F. C. (2016). Human fetal and adult epicardial-derived cells: a novel model to study their activation. *Stem Cell Res. Ther.* 7:174. 10.1186/s13287-016-0434-9 27899163 PMC5129650

[B77] Mol E. A. Goumans M. J. Sluijter J. P. G. (2017). Cardiac progenitor-cell derived exosomes as cell-free therapeutic for cardiac repair. *Adv. Exp. Med. Biol.* 998 207–219. 10.1007/978-981-10-4397-0_14 28936742

[B78] Nemir M. Metrich M. Plaisance I. Lepore M. Cruchet S. Berthonneche C. (2014). The Notch pathway controls fibrotic and regenerative repair in the adult heart. *Eur. Heart J.* 35 2174–2185. 10.1093/eurheartj/ehs269 23166366 PMC4139705

[B79] Nguyen C. T. Dawkins J. Bi X. Marbán E. Li D. (2018). Diffusion tensor cardiac magnetic resonance reveals exosomes from cardiosphere-derived cells preserve myocardial fiber architecture after myocardial infarction. *JACC Basic Transl. Sci.* 3 97–109. 10.1016/j.jacbts.2017.09.005 29600288 PMC5869026

[B80] Pedrotty D. M. Klinger R. Y. Kirkton R. D. Bursac N. (2009). Cardiac fibroblast paracrine factors alter impulse conduction and ion channel expression of neonatal rat cardiomyocytes. *Cardiovasc. Res.* 83 688–697. 10.1093/cvr/cvp164 19477968 PMC2725777

[B81] Pellieux C. Foletti A. Peduto G. Aubert J. F. Nussberger J. Beermann F. (2001). Dilated cardiomyopathy and impaired cardiac hypertrophic response to angiotensin II in mice lacking FGF-2. *J. Clin. Invest.* 108 1843–1851. 10.1172/JCI200113627 11748268 PMC209469

[B82] Perrino C. Barabási A.-L. Condorelli G. Davidson S. M. De Windt L. Dimmeler S. (2017). Epigenomic and transcriptomic approaches in the post-genomic era: path to novel targets for diagnosis and therapy of the ischaemic heart? Position paper of the european society of cardiology working group on cellular biology of the heart. *Cardiovasc. Res.* 113 725–736. 10.1093/cvr/cvx070 28460026 PMC5437366

[B83] Pfeffer M. A. Braunwald E. (1990). Ventricular remodeling after myocardial infarction. *Exp. Observ. Clin. Impl. Circul.* 81 1161–1172. 10.1161/01.cir.81.4.1161 2138525

[B84] Poloni A. Rosini V. Mondini E. Maurizi G. Mancini S. Discepoli G. (2008). Characterization and expansion of mesenchymal progenitor cells from first-trimester chorionic villi of human placenta. *Cytotherapy* 10 690–697. 10.1080/14653240802419310 18985476

[B85] Porrello E. R. Mahmoud A. I. Simpson E. Hill J. A. Richardson J. A. Olson E. N. (2011). Transient regenerative potential of the neonatal mouse heart. *Science* 331 1078–1080. 10.1126/science.1200708 21350179 PMC3099478

[B86] Radosinska J. Bartekova M. (2017). Therapeutic potential of hematopoietic stem cell-derived exosomes in cardiovascular disease. *Adv. Exp. Med. Biol.* 998 221–235. 10.1007/978-981-10-4397-0_15 28936743

[B87] Ribeiro-Rodrigues T. M. Laundos T. L. Pereira-Carvalho R. Batista-Almeida D. Pereira R. Coelho-Santos V. (2017). Exosomes secreted by cardiomyocytes subjected to ischaemia promote cardiac angiogenesis. *Cardiovasc. Res.* 113 1338–1350. 10.1093/cvr/cvx118 28859292

[B88] Sahoo S. Klychko E. Thorne T. Misener S. Schultz K. M. Millay M. (2011). Exosomes from human CD34(+) stem cells mediate their proangiogenic paracrine activity. *Circ. Res.* 109 724–728. 10.1161/CIRCRESAHA.111.253286 21835908 PMC3201702

[B89] Sanada S. Hakuno D. Higgins L. J. Schreiter E. R. McKenzie A. N. J. Lee R. T. (2007). IL-33 and ST2 comprise a critical biomechanically induced and cardioprotective signaling system. *J. Clin. Invest.* 117 1538–1549. 10.1172/JCI30634 17492053 PMC1865027

[B90] Sedrakyan S. Villani V. Da Sacco S. Tripuraneni N. Porta S. Achena A. (2017). Amniotic fluid stem cell-derived vesicles protect from VEGF-induced endothelial damage. *Sci. Rep.* 7:16875. 10.1038/s41598-017-17061-2 29203902 PMC5715019

[B91] Senyo S. E. Steinhauser M. L. Pizzimenti C. L. Yang V. K. Cai L. Wang M. (2013). Mammalian heart renewal by pre-existing cardiomyocytes. *Nature* 493 433–436. 10.1038/nature11682 23222518 PMC3548046

[B92] Shao L. Zhang Y. Lan B. Wang J. Zhang Z. Zhang L. (2017). MiRNA-sequence indicates that mesenchymal stem cells and exosomes have similar mechanism to enhance cardiac repair. *Biomed. Res. Int.* 2017:4150705. 10.1155/2017/4150705 28203568 PMC5292186

[B93] Shivakumar K. Sollott S. J. Sangeetha M. Sapna S. Ziman B. Wang S. (2008). Paracrine effects of hypoxic fibroblast-derived factors on the MPT-ROS threshold and viability of adult rat cardiac myocytes. *Am. J. Physiol. Circ. Physiol.* 294 H2653–H2658. 10.1152/ajpheart.91443.2007 18408121 PMC5875700

[B94] Sluijter J. P. G. Davidson S. M. Boulanger C. M. Buzás E. I. de Kleijn D. P. V. Engel F. B. (2018). Extracellular vesicles in diagnostics and therapy of the ischaemic heart: position paper from the working group on cellular biology of the heart of the european society of cardiology. *Cardiovasc. Res.* 114 19–34. 10.1093/cvr/cvx211 29106545 PMC5852624

[B95] Smart N. Bollini S. Dubé K. N. Vieira J. M. Zhou B. Davidson S. (2011). De novo cardiomyocytes from within the activated adult heart after injury. *Nature* 474 640–644. 10.1038/nature10188 21654746 PMC3696525

[B96] Smith R. R. Barile L. Cho H. C. Leppo M. K. Hare J. M. Messina E. (2007). Regenerative potential of cardiosphere-derived cells expanded from percutaneous endomyocardial biopsy specimens. *Circulation* 115 896–908. 10.1161/CIRCULATIONAHA.106.655209 17283259

[B97] Smits A. M. Dronkers E. Goumans M.-J. (2018). The epicardium as a source of multipotent adult cardiac progenitor cells: their origin, role and fate. *Pharmacol. Res.* 127 129–140. 10.1016/j.phrs.2017.07.020 28751220

[B98] Smits A. M. Van Laake L. W. Den Ouden K. Schreurs C. Szuhai K. Van Echteld C. J. (2009). Human cardiomyocyte progenitor cell transplantation preserves long-term function of the infarcted mouse myocardium. *Cardiovasc. Res.* 83 527–535. 10.1093/cvr/cvp146 19429921

[B99] Takeda N. Manabe I. Uchino Y. Eguchi K. Matsumoto S. Nishimura S. (2010). Cardiac fibroblasts are essential for the adaptive response of the murine heart to pressure overload. *J. Clin. Invest.* 120 254–265. 10.1172/JCI40295 20038803 PMC2798693

[B100] Tauro B. J. Greening D. W. Mathias R. A. Ji H. Mathivanan S. Scott A. M. (2012). Comparison of ultracentrifugation, density gradient separation, and immunoaffinity capture methods for isolating human colon cancer cell line LIM1863-derived exosomes. *Methods* 56 293–304. 10.1016/j.ymeth.2012.01.002 22285593

[B101] Teng X. Chen L. Chen W. Yang J. Yang Z. Shen Z. (2015). Mesenchymal stem cell-derived exosomes improve the microenvironment of infarcted myocardium contributing to angiogenesis and anti-inflammation. *Cell. Physiol. Biochem.* 37 2415–2424. 10.1159/000438594 26646808

[B102] The Lancet Editors (2014). Expression of concern: the SCIPIO trial. *Lancet* 383:1279. 10.1016/S0140-6736(14)60608-5 24725564 PMC5586533

[B103] Théry C. Amigorena S. Raposo G. Clayton A. (2006). Isolation and characterization of exosomes from cell culture supernatants and biological fluids. *Curr. Protoc. Cell Biol.* Chap. 3:Unit 3.22. 10.1002/0471143030.cb0322s30 18228490

[B104] Thum T. Gross C. Fiedler J. Fischer T. Kissler S. Bussen M. (2008). MicroRNA-21 contributes to myocardial disease by stimulating MAP kinase signalling in fibroblasts. *Nature* 456 980–984. 10.1038/nature07511 19043405

[B105] Tian J. Guo X. Liu X. M. Liu L. Weng Q. F. Dong S. J. (2013). Extracellular HSP60 induces inflammation through activating and up-regulating TLRs in cardiomyocytes. *Cardiovasc. Res.* 98 391–401. 10.1093/cvr/cvt047 23447644

[B106] Tseliou E. Fouad J. Reich H. Slipczuk L. De Couto G. Aminzadeh M. (2015). Fibroblasts rendered antifibrotic, antiapoptotic, and angiogenic by priming with cardiosphere-derived extracellular membrane vesicles. *J. Am. Coll. Cardiol.* 66 599–611. 10.1016/j.jacc.2015.05.068 26248985 PMC4593504

[B107] Van Niel G. D’Angelo G. Raposo G. (2018). Shedding light on the cell biology of extracellular vesicles. *Nat. Rev. Mol. Cell Biol.* 19 213–228. 10.1038/nrm.2017.125 29339798

[B108] van Tuyn J. Atsma D. E. Winter E. M. van der Velde-van Dijke I. Pijnappels D. A Bax N. A. (2007). Epicardial cells of human adults can undergo an epithelial-to-mesenchymal transition and obtain characteristics of smooth muscle cells in vitro. *Stem Cells* 25 271–278. 10.1634/stemcells.2006-0366 16990583

[B109] van Vliet P. Smits A. M. de Boer T. P. Korfage T. H. Metz C. H. G. Roccio M. (2010). Foetal and adult cardiomyocyte progenitor cells have different developmental potential. *J. Cell. Mol. Med.* 14 861–870. 10.1111/j.1582-4934.2010.01053.x 20219011 PMC3823117

[B110] Vrijsen K. R. Maring J. A. Chamuleau S. A. J. Verhage V. Mol E. A. Deddens J. C. (2016). Exosomes from cardiomyocyte progenitor cells and mesenchymal stem cells stimulate angiogenesis Via EMMPRIN. *Adv. Healthc. Mater.* 5 2555–2565. 10.1002/adhm.201600308 27570124

[B111] Waldenström A. Gennebäck N. Hellman U. Ronquist G. (2012). Cardiomyocyte microvesicles contain DNA/RNA and convey biological messages to target cells. *PLoS One* 7:e34653. 10.1371/journal.pone.0034653 22506041 PMC3323564

[B112] Wang H.-S. Hung S.-C. Peng S.-T. Huang C.-C. Wei H.-M. Guo Y.-J. (2004). Mesenchymal stem cells in the wharton’s jelly of the human umbilical cord. *Stem Cells* 22 1330–1337. 10.1634/stemcells.2004-0013 15579650

[B113] Wang Y. Chiu A. P. Neumaier K. Wang F. Zhang D. Hussein B. (2014). Endothelial cell heparanase taken up by cardiomyocytes regulates lipoprotein lipase transfer to the coronary lumen after diabetes. *Diabetes Metab. Res. Rev.* 63 2643–2655. 10.2337/db13-1842 24608441

[B114] Winter E. M. Grauss R. W. Hogers B. Van Tuyn J. Van Der Geest R. Lie-Venema H. (2007). Preservation of left ventricular function and attenuation of remodeling after transplantation of human epicardium-derived cells into the infarcted mouse heart. *Circulation* 116 917–927. 10.1161/CIRCULATIONAHA.106.668178 17684151

[B115] Wong L. L. Armugam A. Sepramaniam S. Karolina D. S. Lim K. Y. Lim J. Y. (2015). Circulating microRNAs in heart failure with reduced and preserved left ventricular ejection fraction. *Eur. J. Heart Fail.* 17 393–404. 10.1002/ejhf.223 25619197

[B116] Yarana C. Carroll D. Chen J. Chaiswing L. Zhao Y. Noel T. (2018). Extracellular vesicles released by cardiomyocytes in a doxorubicin-induced cardiac injury mouse model contain protein biomarkers of early cardiac injury. *Clin. Cancer Res.* 24 1644–1653. 10.1158/1078-0432.CCR-17-2046 29070527 PMC6193451

[B117] Yu B. Kim H. W. Gong M. Wang J. Millard R. W. Wang Y. (2015). Exosomes secreted from GATA-4 overexpressing mesenchymal stem cells serve as a reservoir of anti-apoptotic microRNAs for cardioprotection. *Int. J. Cardiol.* 182 349–360. 10.1016/j.ijcard.2014.12.043 25590961 PMC4382384

[B118] Zhang H. Freitas D. Kim H. S. Fabijanic K. Li Z. Chen H. (2018). Identification of distinct nanoparticles and subsets of extracellular vesicles by asymmetric flow field-flow fractionation. *Nat. Cell Biol.* 20 332–343. 10.1038/s41556-018-0040-4 29459780 PMC5931706

[B119] Zhang Z. Yang J. Yan W. Li Y. Shen Z. Asahara T. (2016). Pretreatment of cardiac stem cells with exosomes derived from mesenchymal stem cells enhances myocardial repair. *J. Am. Heart Assoc.* 5:e002856. 10.1161/JAHA.115.002856 26811168 PMC4859399

[B120] Zhao L. Borikova A. L. Ben-Yair R. Guner-Ataman B. MacRae C. A. Lee R. T. (2014). Notch signaling regulates cardiomyocyte proliferation during zebrafish heart regeneration. *Proc. Natl. Acad. Sci. U.S.A.* 111 1403–1408. 10.1073/pnas.1311705111 24474765 PMC3910613

[B121] Zhao Y. Sun X. Cao W. Ma J. Sun L. Qian H. (2015). Exosomes derived from human umbilical cord mesenchymal stem cells relieve acute myocardial ischemic injury. *Stem Cells Int.* 2015:761643. 10.1155/2015/761643 26106430 PMC4461782

[B122] Zhou B. Honor L. B. He H. Ma Q. Oh J.-H. Butterfield C. (2011). Adult mouse epicardium modulates myocardial injury by secreting paracrine factors. *J. Clin. Invest.* 121 1894–1904. 10.1172/JCI45529 21505261 PMC3083761

[B123] Zhou B. Pu W. T. (2011). Epicardial epithelial-to-mesenchymal transition in injured heart. *J. Cell. Mol. Med.* 15 2781–2783. 10.1111/j.1582-4934.2011.01450.x 21914126 PMC3226901

[B124] Zhu J. Lu K. Zhang N. Zhao Y. Ma Q. Shen J. (2017). Myocardial reparative functions of exosomes from mesenchymal stem cells are enhanced by hypoxia treatment of the cells via transferring microRNA-210 in an nSMase2-dependent way. *Artif. Cells Nanomed. Biotechnol.* 10.1080/21691401.2017.1388249 [Epub ahead of print]. 29141446 PMC5955787

[B125] Zwetsloot P. P. Végh A. M. D. Jansen of Lorkeers S. J. van Hout G. P. J. Currie G. L. Sena E. S. (2016). Cardiac stem cell treatment in myocardial infarctionnovelty and significance. *Circ. Res.* 118 1223–1232. 10.1161/CIRCRESAHA.115.307676 26888636

